# Effects of Growth Hormone on Cardiac Remodeling During Resistance Training in Rats

**DOI:** 10.5935/abc.20160003

**Published:** 2016-01

**Authors:** Adriana Junqueira, Antônio Carlos Cicogna, Letícia Estevam Engel, Maiara Almeida Aldá, Loreta Casquel de Tomasi, Rogério Giuffrida, Inês Cristina Giometti, Ana Paula Coelho Figueira Freire, Andreo Fernando Aguiar, Francis Lopes Pacagnelli

**Affiliations:** 1 Universidade do Oeste Paulista (UNOESTE), Presidente Prudente, SP - Brazil; 2 Universidade Estadual Paulista (UNESP), Campus Botucatu, SP - Brazil; 3 Universidade Estadual Paulista (UNESP), Campus Presidente Prudente, SP - Brazil; 4 Universidade do Norte do Paraná, UNOPAR, Londrina, PR - Brazil

**Keywords:** Growth Hormone, Rats, Motor Activity, Exercise, Ventricular Remodeling

## Abstract

**Background:**

Although the beneficial effects of resistance training (RT) on the cardiovascular system are well established, few studies have investigated the effects of the chronic growth hormone (GH) administration on cardiac remodeling during an RT program.

**Objective:**

To evaluate the effects of GH on the morphological features of cardiac remodeling and Ca2+ transport gene expression in rats submitted to RT.

**Methods:**

Male Wistar rats were divided into 4 groups (n = 7 per group): control (CT), GH, RT and RT with GH (RTGH). The dose of GH was 0.2 IU/kg every other day for 30 days. The RT model used was the vertical jump in water (4 sets of 10 jumps, 3 bouts/wk) for 30 consecutive days. After the experimental period, the following variables were analyzed: final body weight (FBW), left ventricular weight (LVW), LVW/FBW ratio, cardiomyocyte cross-sectional area (CSA), collagen fraction, creatine kinase muscle-brain fraction (CK-MB) and gene expressions of SERCA2a, phospholamban (PLB) and ryanodine (RyR).

**Results:**

There was no significant (p > 0.05) difference among groups for FBW, LVW, LVW/FBW ratio, cardiomyocyte CSA, and SERCA2a, PLB and RyR gene expressions. The RT group showed a significant (p < 0.05) increase in collagen fraction compared to the other groups. Additionally, the trained groups (RT and RTGH) had greater CK-MB levels compared to the untrained groups (CT and GH).

**Conclusion:**

GH may attenuate the negative effects of RT on cardiac remodeling by counteracting the increased collagen synthesis, without affecting the gene expression that regulates cardiac Ca^2+^ transport.

## Introduction

The use of growth hormone (GH) as an ergogenic aid has risen dramatically in the past two decades, especially among athletes involved in strength, hypertrophy and power trainings (bodybuilders and weightlifters) and recreational practitioners interested in maintaining good health and enhancing the physique. However, GH use as an ergogenic aid for athletes is forbidden by the World Anti-Doping Agency (WADA),^1^ because of its direct effects on performance, such as body fat reduction, strength and muscle mass increase, and skeletal muscle regeneration. In addition, the inappropriate use of GH can lead to a decline in performance and irreparable health damage.

Growth hormone can affect heart functioning and cause cardiac hypertrophy, without fibrosis increase.^2,3^ This response is accompanied by an increase in contractility, changes in the genesis of the cardiac potential of action and peripheral vasodilation.^2,3^ Some studies have shown the cardioprotective effect of GH after myocardial infarction, easing pathological cardiac remodeling.^2^ However, other studies have reported the damage caused by the chronic hypersecretion of GH (acromegaly), leading to the development of concentric cardiac hypertrophy with interstitial fibrosis and lymphomononuclear infiltrate. If not controlled, the elevated GH level can lead to heart failure.^3,4^ Although other risk factors are related to acromegaly, the excess of GH and of its mediator [insulin-like growth factor 1 (IGF-1)] might be the major contributor to cardiovascular disease.^4^

Growth hormone has been often used to increase muscle mass and strength, and to enhance cardiac function during programs of resistance training (RT). Although the beneficial effects of RT on the cardiovascular system, such as increased capillary density, left ventricular (LV) hypertrophy, changes in connective tissue and benefits to the cardiac function, have been well established,^5,6^ few studies have investigated the effects of chronic GH administration on cardiac remodeling during RT programs.

This study aimed at testing the hypothesis that GH administration during RT modulates cardiac remodeling, interfering with morphological parameters and the gene expression of proteins involved in Ca^2+^ homeostasis, such as sarcoplasmic reticulum calcium-ATPase (SERCA2a) pump, phospholamban (PLB) and ryanodine (RyR). The gene expression of SERCA2a, PLB and RyR was analyzed because of its important role in cardiac contractile function, acting on intracellular Ca^2+^ homeostasis.^7^

## Methods

### Animals and procedures

This study assessed 28 male Wistar rats (mean weight of 235 ± 15.2 g) aged 9 weeks-old, from the Central Vivarium of the Oeste Paulista University (UNOESTE), São Paulo, Brazil. The animals were individually labeled, and housed in seven cages containing four animals each, with free access to water and food (SupraLab®). The standard environmental conditions were maintained, with controlled light (12-hour light/dark cycles, with light from 7 AM on), temperature (21 ± 5°C) and relative air humidity (55% ± 5%).

This study was approved by the Committee on Animal Research and Ethics of the UNOESTE (protocols 1688 and 1689), and abided by the *Guide for the Care and Use of Laboratory Animals*, published by the National Research Council.

### Study design

After an adaptation of seven days, the rats were distributed into four groups: control (CT, n = 7); receiving GH (GH, n = 7); undergoing RT (RT, n = 7); and association of RT and GH administration (RTGH, n = 7).

### GH administration

The GH group animals received 0.2 IU/kg of recombinant human GH (rhGH, Saizen® - Merck) subcutaneously, every 2 days, for 30 consecutive days.^8^ The other animals received a similar volume of saline solution (0.9% NaCl).

### Resistance training

Physical training was performed according to a protocol of vertical jumps in water, 3 times per week, for 30 consecutive days. One week before starting the experiment, the rats were adapted to water exercise, with a daily increase in the number of sets and weight load of 50% of their total body weight. The training occurred inside a PVC tube (diameter of 25 cm, and depth of 38 cm), filled with warm water (30°C) as described by De Mello Malheiro et al.^9^ After the adaptation period, the animals initiated the training protocol, each session consisting of 4 sets of 10 jumps at 1-minute rest intervals between the sets. The rats were weighed before each session to recalculate their weight load (50% of the total body weight). The overload consisted in wearing a Velcro vest with fixed weights in the anterior region of the chest. At the end of each training, the animals were dried and returned to their cages.

### Parameters analyzed

By the end of 4 weeks, 72 hours after the last training session, the animals were weighed and sacrificed by exsanguination under anesthesia with ethylic ether. Their hearts were removed and weighed, the left ventricle dissected, and weighed again. The LV apex was frozen in liquid nitrogen, while the LV upper portion was fixed in 10% formalin solution for gene expression and morphological studies, respectively. The humid LV weight (LVW), normalized to the rat’s final body weight (FBW), was used as an index of ventricular hypertrophy (LVW/FBW ratio).

### Morphological study

Samples of cardiac tissue were fixed in 10% formalin solution for 48 hours. After fixation, the tissue was embedded in paraffin blocks, and 4-micrometer histological sections obtained and mounted in glass slides. The histological sections were stained with Hematoxilin-Eosin (HE) to assess the cross-sectional area of cardiomyocytes, by using a LEICA DM750 microscope coupled to a video camera that sends digital images to a computer, which has a program for image analysis (Image Pro-plus, Media Cybernetics, Silver Spring, Maryland, USA). The images were obtained by use of a binocular optical microscope. All images were captured by the video camera at the 40x magnification. Image selection for capture and digitalization was performed visually. All analyses were performed by one single examiner, blinded to the group of images. The morphometry of those images obtained and digitalized was performed by using appropriate software. Four LV sections were obtained for each animal. In each section, different fields were captured, chosen according to the site exhibiting the highest number of cells on a cross‑section. For each ventricle analyzed, 50 cells were measured. The myocytes selected were cross-sectioned, had a round shape and visible central nucleus, and were located in the subendocardial layer of the LV muscular wall. This was aimed at standardizing the set of myocytes of the different groups. The mean cardiomyocyte cross-sectional areas obtained for each group were used as indicators of cell size.^10^

Six-micrometer coronal histological sections were mounted in glass slides and stained with Picrosirius Red, specific for collagen visualization, to assess the LV myocardial interstitium. Collagen fibers were visualized in red and myocytes in yellow. The collagen volume fraction as a percentage was automatically calculated and corresponded to the sum of the collagen areas divided by the sum of the collagen tissue area and the cardiomyocyte cross-sectional area. The images of the cardiac tissue were captured by a LEICA DM LS microscope coupled to a video camera that sends digital images to a computer, which has a program for image analysis (Image Pro‑plus, Media Cybernetics, Silver Spring, Maryland, USA). For each ventricle, 20 fields were analyzed, using a 40X objective. The fields far away from the perivascular region were chosen.

### Gene expression of intracellular Ca^2+^ regulators

Total RNA was extracted from the cardiac tissue (left ventricle) by using TRIzol Reagent (Invitrogen), and then treated with DNAse according to the manufacturer’s recommendation. Electrophoresis was used to assess RNA integrity. The High Capacity cDNA Reverse Transcription kit (Applied Biosystems, CA, USA) was used to synthesize complementary DNA (cDNA) from 1000 ng of total RNA. RT-qPCR was used to quantitatively measure the relative levels of mRNA from SERCA2a (Rn00568762_m1), RyR (Rn01470303_m1) and PLB (Rn01434045_m1). TaqMan Universal PCR Master Mix (Applied Biosystems, CA, USA) was used according to the manufacturer’s recommendation, as was the Applied Biosystems StepOne Plus detection system. All samples were assessed twice. The cycling conditions were as follows: enzyme activation at 50°C for 2 minutes; denaturation at 95°C for 10 minutes; amplification of cDNA products with 40 denaturation cycles at 95°C for 15 seconds; and annealing/extension at 60°C for 1 minute. Gene expression was quantified in relation to the CT group values and after normalization with β-actin as an internal control (ACTB, Rn00667869_m1), and determined according to the 2-ΔΔCt method, as previously described.^11^

### Measuring CK-MB

Blood was collected for serum biochemistry of creatine kinase muscle-brain fraction (CK-MB) in tubes (Vacutainer®) without anticoagulant. After that, total blood was centrifuged at 3000 rpm (g = 1257). The serum obtained was stored in plastic microtubes and maintained at -20°C. The test was performed with the automatic UV-Kinetic method (Cobas C111, Roche®).

### Data analysis

To compare the parameters studied between the experimental groups and to validate the assumptions of data normality and homogeneity of variance, the Shapiro-Wilk and Levene’s tests were performed, respectively. Data with normal distribution underwent one-way analysis of variance (one-way ANOVA) and Tukey’s test for contrasts, while the Kruskal-Wallis test was used for data with non-normal distribution. Parametric variables were expressed as mean ± standard deviation, and non-parametric as median and 25^th^ and 75^th^ percentiles. The SPSS software for Windows, version13.0, was used in all analyses, and the statistical significance level adopted was 5%.

## Results

**Figure 1:**
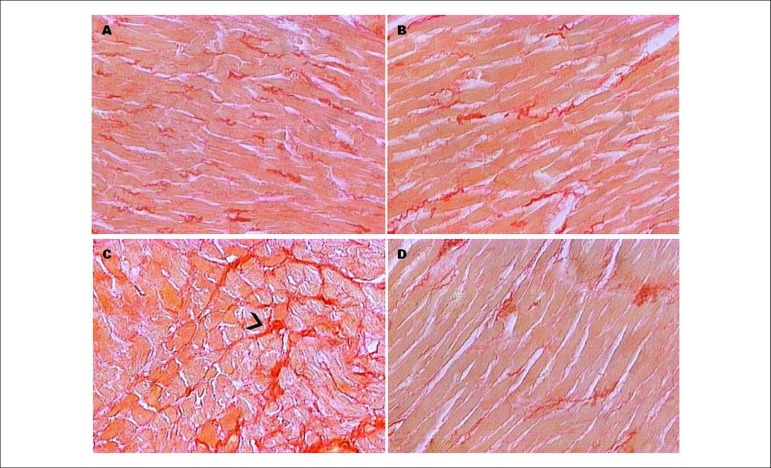
Technique of myocardial collagen staining: Picrosirius Red. Optical microscope, 40X magnification. Collagen stained in red. A - control group (CT); B - growth hormone group (GH); C - resistance training group (RT); D - resistance training and growth hormone group (RTGH).

**Figure 2:**
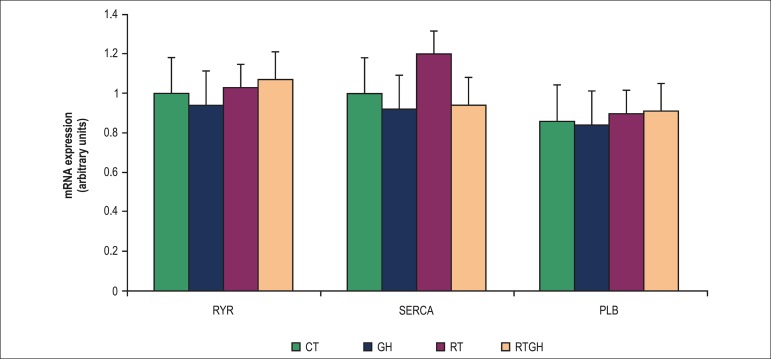
Relative levels of mRNA determined by qPCR of ryanodine (RyR), sarcoplasmic reticulum calcium-ATPase (SERCA2a) and phospholamban (PLB), expressed as mean ± standard deviation. CT: Control group; GH: Growth hormone group; RT: Resistance training group; RTGH: Resistance training + growth hormone group.

**Figure 3:**
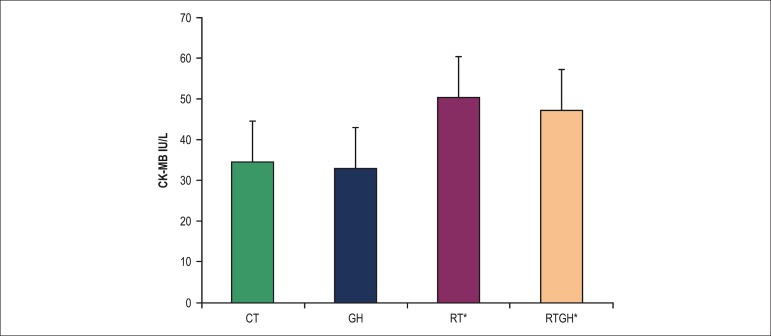
Measurement of creatine kinase muscle-brain fraction (CK-MB) by use of biochemical analysis, expressed as mean ± standard deviation. CT: Control group; GH: Growth hormone group; RT: Resistance training group; RTGH: Resistance training + growth hormone group. *p < 0.05 (statistically significant difference): CT versus RT; p < 0.05 CT versus RTGH; p < 0.05 GH versus RT; p < 0.05 GH versus RTGH.

Table 1 and Figure 1 show the anatomic and morphological parameters indicating cardiac remodeling, and Figure 2 shows data on gene expression. The FBW, LVW, LVW/FBW ratio and cardiomyocyte cross-sectional area (Table 1) showed no statistical difference (p > 0.05), and neither has the gene expression of the regulatory proteins (RyR, SERCA2a and PLB) (Figure 2). However, the RT group showed a significant increase (p < 0.05) in the interstitial collagen fraction as compared to all other groups (Table 1). That increase did not occur when RT was combined with GH (RTGH group) (Table 1). In addition, the groups trained (RT and RTGH) showed higher CK-MB levels as compared to the non-trained ones (CT and GH) (Figure 3).

**Table 1:** Anatomical parameters (weight) expressed as mean ± standard deviation, median and 25^th^ and 75^th^ percentiles, and cardiomyocyte cross-sectional area and interstitial collagen fraction expressed as mean ± standard deviation

**Variables**	**Groups**			
	**CT**	**GH**	**RT**	**RTGH**
FBW (g)	297.27 ± 22.14	313.81 ± 14.02	304.30 ± 29.08	292.05 ± 15.96
	305.10	309	307.10	285.30
	[286.90 - 315.00]	[300.40 - 332.40]	[286.5 - 332.00]	[273.40 - 332.40]
LVW (g)	0.74 ± 0.31	0.87 ± 0.25	0.75 ± 0.26	0.77 ± 0.23
	0.61	0.74	0.67	0.68
	[0.53 - 1.16]	[0.62 - 1.14]	[0.43 - 0.73]	[0.60 - 0.98]
LVW/FBW (mg/g)	2.48 ± 0.95	2.76 ± 0.79	2.55 ± 1.04	2.64 ± 0.81
	2.04	2.35	2.12	2.38
	[1.85 - 3.80]	[2.07 - 3.53]	[1.27 - 2.38]	[2.03 - 3.35]
CSA (µm^2^)	343.64 ± 56.67	364.06 ± 48.31	412.84 ± 78.50	344.44 ± 35.43
IC (%)	2.55 ± 1.10	2.19 ± 0.70	5.74 ± 2.62	2.54 ± 0.65

CT: No exercise and no growth hormone (control); GH: No exercise + growth hormone; RT: Resistance training; RTGH: Resistance training + growth hormone; FBW: Final body weight; LVW: Left ventricular weight; CSA: Cardiomyocyte cross-sectional area; µm^2^: Micrometer; IC: Interstitial collagen fraction; g: Grams. * p < 0.05 vs. CT, GH, RTGH groups.

## Discussion

This study aimed at investigating the effects of GH administration, isolated or combined with RT, on the morphological parameters and gene expression of the major proteins involved with Ca^2+^ transport (SERCA2a, PLB and RyR) during cardiac remodeling. This study’s findings were: 1) RT isolated acted on cardiac remodeling, increasing LV collagen density; and 2) GH administration during RT modulates cardiac remodeling, attenuating the increase in collagen fraction, without altering myocardial damage and the proteins involved with Ca^2+^ transport.

The RT group showed higher collagen synthesis then the other groups. That could be explained by the cardiac pressure overload imposed by the mechanical stress of RT, which could have induced collagen breakdown, thus stimulating collagen synthesis.^12,13^ That hypothesis is supported by a study showing an increase in interstitial collagen formation induced by aerobic training or RT.^4^ The study by De Souza et al.^5^ has shown a 2.8% increase in the interstitial collagen fraction in the group submitted to RT (control group = 5.5% and trained group = 9%). In our study, that increased from 2.55% to 5.74%. Our collagen fraction values are not in accordance with those of the study by De Souza et al.,^5^ because the age and weight of the animals were different. However, other studies by our team with male Wistar rats of similar weights corroborate our findings, in which the collagen fraction varied from 2.5% to 3% in the control group.^14,15^ Our results expand the previous observations, showing for the first time that an increase in collagen synthesis can occur during RT with no change in the cardiomyocyte cross-sectional area. That effect can be attributed, at least partially, to the cardiomyocyte damage^16^ induced by physical exercise,^17-19^ as demonstrated in our study by the elevation in CK-MB levels.

Curiously, the increase in collagen synthesis was attenuated when GH was combined with RT. The result is consistent with previous studies showing a cardioprotective effect of GH on the synthesis of type I and III collagen during pathological cardiac remodeling.^20,21^ In rats with ventricular hypertrophy induced by chronic pressure overload, GH induced a cardioprotective effect, attenuating myocardial fibrosis.^21^ However, not analyzing the collagen type (IαI, IαII and III) was a limitation of our study, because it can vary according to the type of stimulus (physiological or pathological), and with the supplementation of substances and exercise intensity. Thus, further studies are necessary to determine the collagen type and the molecular pathways that are activated in response to different exercise types.

Despite the increase in collagen synthesis, no difference was observed in the cardiomyocyte cross-sectional areas or in the LVW/FBW ratio following RT isolated or combined with GH, demonstrating that no cardiac hypertrophy occurred. Our cardiomyocyte cross-sectional area values (CT group: mean of 343.64µ^2^) are in accordance with those of another study also using male Wistar rats of similar weights (control group: 305.6 - 333µ^2^).^15^

Those results are not in accordance with the results of other studies involving different overload stimuli.^21-26^ Moreira et al.^21^ have not found changes in the LWE/FBW ratio of rats submitted to chronic pressure overload in an aortic stenosis model after a short period of treatment with GH (1 mg/kg for 14 days). Those authors have only shown myocardial fibrosis attenuation as a cardioprotective effect, indicating a specific effect of that hormone. In addition, Sugizaki et al.^22^ have not evidenced differences in the LVW/FBW ratio in rats swimming with weight overload (5% of body weight). The animals were submitted to five swimming sessions per week, for 12 consecutive weeks. However, Baraúna et al.^23^ have reported an increase in the diameter of cardiomyocytes of rats submitted to an RT protocol consisting in upper body extension (with maximum lifted weight with the exercise apparatus), 4 sets of 12 repetitions, using 65% to 75% of one repetition maximum, for 4 or 12 weeks.

That type of training induced concentric cardiac hypertrophy with neither ventricular dysfunction nor cavity reduction. The reasons for the conflicting results are not clear, but can be due to the training protocol (period, intensity, volume) and GH doses used.^23,26^

The exact cellular and molecular mechanisms of the reported effects of GH and RT on the heart have not been completely elucidated.^23,24,26^ In this study, no change in the genes related to cardiac Ca^2+^ transport was observed. The cardiomyocytes express receptors for the secretion of GH and IGF-1, and such receptors are influenced by hemodynamic changes. Both hormones are believed to have stimulating effects on myocardial contractility.^26^ In addition, those hormones and GH-releasing peptides, such as ghrelin, have beneficial cardiac effects.^26-28^ Ma et al.^7^ have reported that the activation of the GHS-R1a ghrelin receptor produced an inotropic effect on ischemic cardiomyocytes resulting from ischemia/reperfusion injury, because they might protect or recover Ca^2+^ transport proteins, such as SERCA2a and PLB. In this study, we expected that the effect on cardiac remodeling could be mediated by the elevation in the expression of those genes, resulting from GH administration, because that hormone boosts the cell protein synthesis and mRNA formation. However, that did not occur.

Two possible physiological explanations for that fact could be the lower sensitivity of the cardiovascular tissue to the direct effect of GH,^24^ and the negative feedback of GH and IGF due to their exogenous administration, which could have interfered in the regulation of GH synthesis and secretion.^27-29^ In addition, the lack of GH effects on RT and the genes of Ca^2+^ transport can be due to the training protocol. Previous studies have already reported the change in gene expression of cardiac Ca^2+^ after a training program of 8 weeks.^29-32^ Although RT increases the intracellular Ca^2+^ concentration, increasing cardiomyocyte contractility and overexpression of SERCA2a, and, consequently, of its regulator PLB,^30,31^ our results showed no change in the variables assessed in the time period assessed.

## Conclusions

The RT caused cardiac interstitial collagen remodeling without changes in cardiomyocyte cross-sectional areas. That, however, did not occur when the RT was associated with GH administration. The results indicate that GH can attenuate the effects of RT on cardiac remodeling by counterbalancing the increase in collagen synthesis, without affecting the expression of the genes that regulate cardiac Ca^2+^ transport.
